# Laboratory Evaluation of the Performance of Stone Mastic Asphalt as an Ungrooved Runway Surface

**DOI:** 10.3390/ma14030502

**Published:** 2021-01-21

**Authors:** Sean Jamieson, Greg White

**Affiliations:** Airport Pavement Research Program, School of Science, Technology and Engineering, University of the Sunshine Coast, Sippy Downs, QLD 4556, Australia; sean.jamieson@research.usc.edu.au

**Keywords:** runway, asphalt, ungrooved, stone mastic, stone mastic asphalt (SMA)

## Abstract

Many airports are surfaced with grooved Marshall-designed dense graded asphalt. Grooving is required to satisfy regulatory aircraft skid resistance requirements, but introduces the risk of groove-related distress, such as groove closure. Consequently, airports seek an ungrooved runway surface option that performs similarly to dense graded asphalt but allows grooving to be avoided. Stone mastic asphalt is the most viable ungrooved runway surface solution and has been used on runways in Europe and China. However, before being accepted as an ungrooved runway surface in Australia, stone mastic asphalt must be shown to meet regulatory runway aircraft skid resistance requirements, and to otherwise perform similarly to typical dense graded asphalt mixtures for runway surfacing, including deformation resistance, fatigue cracking resistance and durability. Based on laboratory performance-related testing, 10-mm and 14-mm sized stone mastic asphalt mixtures, produced with four different aggregate sources, were found to generally meet the airport asphalt performance requirements. The 14 mm mixture was found to perform better than the 10 mm mixture, particularly regarding surface macrotexture and deformation resistance. It was concluded that airports should consider 14 mm sized stone mastic asphalt as an ungrooved runway surface in the future.

## 1. Introduction

In Australia and some other countries, runways are commonly surfaced with grooved Marshall-designed dense graded asphalt (DGA). Grooving is required to enable the runway to shed water during wet weather events, as well as satisfying regulatory requirements set by the International Civil Aviation Authority (ICAO) for aircraft skid resistance, which are mandated in Australia by the Civil Aviation Safety Authority (CASA) [1]. Groove closure is a runway surface distress that inhibits the drainage ability of the runway surface [2] and reduces the friction characteristics of the pavement surface during wet weather, increasing the likelihood of hydroplaning [3]. In addition, when groove closure does occur, the cost of rectification is substantial, as is the impact on the operational capacity of the runway.

There are alternates to grooved DGA that are used in some counties for runway surfacing. Of these materials, stone mastic asphalt (SMA) is commonly used due to its high rut resistance and coarse macrotexture, negating the need for grooving [4]. SMA is used on Australian roads as a premium surfacing for heavy-duty traffic. However, the use of SMA on Australian airport pavements is limited. Australian airport operators lack the confidence required to use SMA because this asphalt surface material has neither been translated into Australian airport specifications, nor has its performance been validated as an Australian airport surface material. To validate SMA as a suitable alternate runway surface, it must achieve the published and established airport asphalt performance requirements. That is, the performance of ungrooved SMA must be no worse than for comparable grooved DGA. The performance requirements include deformation resistance and fracture resistance, as well as mixture durability [5]. Furthermore, to allow grooving to be avoided, SMA must also meet the regulatory requirements for aircraft skid resistance, including surface friction and surface texture.

This research evaluated the laboratory performance of SMA as an ungrooved runway surface. Firstly, the performance requirements expected of runway wearing courses are discussed, with an emphasis on skid resistance and surface texture. Then the process of evaluating SMA against these requirements is outlined. Finally, the results from a laboratory evaluation of seven SMA mixtures are presented and discussed in order to determine the suitability of SMA as an ungrooved runway surface. Although focused on the Australian context and using Australian test methods, this research is also valid for the United Kingdom (UK), the United States of America (USA), the Middle East and other countries that generally use grooved DGA for runway surfacing.

## 2. Background

### 2.1. Airport Asphalt

In Australia, the USA and the UK, Marshall-designed DGA is the predominant surfacing material for flexible sealed aircraft pavements [2]. The Marshall mixture design method was developed by the United States Department of Defense between World War II to the late 1950s, to design and control asphalt mixtures [6]. The Marshall method involves compacting a laboratory sample with a prescribed number of blows of a standard (Marshall) hammer, intended to simulate the expected in-field traffic conditions. An optimum bituminous binder content is then selected based on laboratory measured properties known as the Marshall Stability and the Marshall Flow, as well as the unit weight of the total mix, percentage of voids in the total mix and the percentage of the voids (in the aggregate) that is filled with binder.

In Australia, a 14-mm sized Marshall-designed airport DGA usually requires 13–17% (by volume) voids in the mineral aggregate (VMA) and an air void content of 3.5–4.5% [7]. Typically, a 5.4–5.8% (by mass) bituminous binder content is required to achieve this [8].

### 2.2. Performance Requirements

Traditionally, airfield asphalt was specified based on a prescriptive or recipe-based approach [8]. These requirements focus on achieving a target aggregate gradation, Marshall properties and volumetric properties of samples prepared using the Marshall method. However, since the development of the Marshall method, aircraft have become heavier, with significantly higher tyre pressures. Coupled with evidence of a reduction in binder quality in Australia [9], some airport asphalt surfaces that were compliant with the prescriptive requirements have failed to perform as expected in the field [2].

The reduced confidence in the traditional prescriptive-based specification has led to the development of a performance-related specification of Australian airport asphalt [8]. This performance-related specification was first released in February 2018 and focuses on four main physical requirements intended to protect the pavement surface from common distresses. In the performance-related specification, standardised tests are used to be able to assess each of the physical performance requirements during mixture design [7]. The performance-indicative testing provides confidence and objective evidence that the mixture can perform as expected in the field, prior to the commencement of any asphalt paving. Table 1 details the performance requirements, as well as the standardised tests used in the Australian airport DGA performance-related specification [7]. The performance-indicative testing within the specification provides the basis upon which any alternate (to DGA) asphalt mixture, or type, can be evaluated. That is, if an alternate asphalt type satisfies the performance requirements in the specification, it is theoretically considered to be fit for purpose as a runway surfacing.

### 2.3. Skid Resistance and Grooving

It is important to note that Table 1 does not include any test for surface friction or texture. This is because 14-mm sized DGA has insufficient surface texture to meet the regulatory requirements, as well as because testing for surface friction can only be performed reliably in the field, after construction is complete. Therefore, no performance-related test is nominated during mixture design. However, skid resistance is still important, with the Australian regulation, known the Manual of Standards 139 (MOS 139), requiring every runway, regardless of size, to satisfy at least one of the following [1]:

- 1-mm surface texture, or;
- minimum friction levels measured by continuous friction measuring equipment (CFME).

Airport DGA surfaces will not achieve the 1-mm requirement and will typically have a surface texture of 0.4–0.6 mm [10]. Additionally, the initial wetted surface friction of DGA, measured using CFME, can be marginal when compared to regulatory values [11]. Therefore, to meet the regulatory skid resistance requirement, and to mitigate the risk of hydroplaning during wet weather, DGA runway surfaces are commonly grooved, which increases the CFME measured friction levels to above the values required.

Grooving a runway surface is expensive, disruptive, complicates pavement maintenance and introduces the risk of groove-related distress. Groove closure is one the most commonly reported airport asphalt surface distresses in hot climates and impacts the ability of the pavement to shed surface water, due to the reduction in volume of the grooves [2]. Repairing grooves by re-sawing is not possible, and the only solution is to remove the closed grooves, replace with new asphalt, and regroove the new surface [12]. Not only is this process costly, it also impacts the operation of the airport during the repair work. Consequently, airports desire an alternate asphalt mixture that achieves skid resistance requirements without the need to groove. Of the alternates available, SMA is likely to be the most appropriate [5]. However, for ungrooved SMA to be validated as a suitable runway surface, it must have laboratory performance comparable to airport DGA, as well as having a surface texture greater than 1 mm and/or surface friction levels above the regulatory minimums.

### 2.4. Stone Mastic Asphalt as an Alternate Surface

SMA was originally developed in Germany in the 1960s. The aim was to reduce distress in asphalt wearing courses caused by studded snow tyres [13]. Like all asphalt mixtures, SMA consists of three parts:

- a coarse aggregate skeleton;
- a bituminous mastic;
- the air voids.

**Figure 1:**
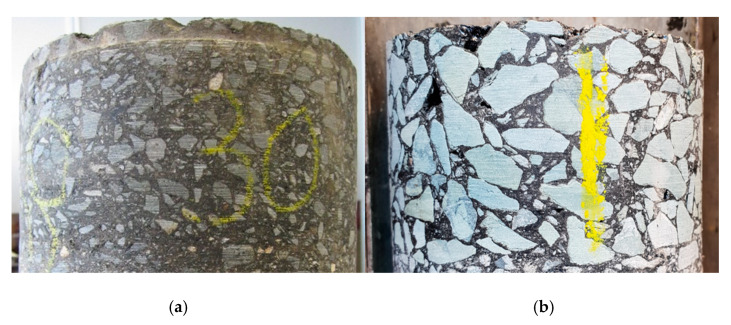
Core samples of (**a**) dense graded asphalt (DGA) and (**b**) stone mastic asphalt (SMA).

The coarse aggregate skeleton is composed of aggregate larger than what is known as the break-point sieve (4.75 mm for 10–14 mm size mixtures) and provides the high deformation resistance due to stone-on-stone contact (Figure 1). At the same time, a high binder and mastic content (filler, fines and binder) results in a durable and fatigue-resistant mixture. However, the higher binder content also introduces the risk of binder drain down during production, transportation and paving. To mitigate binder drain down, SMA mixtures will usually include stabilisers, or drainage inhibitors, commonly in the form of cellulose fibers.

Critically important to the use of SMA as an ungrooved runway surface is the surface macrotexture. Due to its gap-graded nature, SMA mixtures larger than 10 mm maximum aggregate size usually exhibit texture depths greater than 1 mm [14,15,16], which satisfies the ICAO and CASA runway skid resistance requirements.

Despite its limited use in Australia, SMA has been used as a runway surface in Europe and China, with surface trials also undertaken in South Africa and the USA [17,18]. Norway has used SMA as a runway surface on more than 15 runways since 1992 [18], including the western runway of Oslo international airport, which was resurfaced with an 11-mm sized SMA in 2015 [19]. Germany has also used an 11-mm sized SMA for runway surfacing at Hamburg airport and Spangdahlem Air Force Base [17] and three runways surfaced with SMA at Frankfurt airport. Other European countries that have used SMA as a runway surfacing include Spain, the UK, Italy, Austria, Denmark and Sweden. China, however, is the global leader of SMA use on airfields with over 40 airports, including Beijing and Shanghai, surfaced with either 13-mm or 16-mm sized mixtures [20].

The successful use of SMA as a runway surface in Europe and China, and its routine use on Australian roads, has indicated that SMA is likely suitable as an ungrooved runway surface in Australia. However, for SMA to be accepted by Australian airports, particularly for runway surfacing, it must be evaluated against the laboratory performance requirements expected of airport runway asphalt, as well as the regulatory requirements for friction and surface texture. Binder drain down requirements must also be demonstrated.

### 2.5. Runway Asphalt Surface Requirements

In 2017, performance requirements for DGA used as an airport pavement surface were developed in Australia [8]. A performance-related specification was subsequently developed [7]. The specification largely retains the traditional DGA volumetric requirements but allows the mixture designer to select or develop any bituminous binder to achieve the specified performance properties (Table 2). In 2019 the Australian airport performance-related specification for DGA was expanded to include the requirements of SMA [14]. The incorporation of SMA into the specification required:

- minor changes to aggregate property requirements for reliable SMA production;
- adding SMA volumetric composition requirements for mixture design;
- removal and adjustment of inappropriate DGA test methods;
- adding volumetric surface texture and binder drain down tests to SMA mixture design, and;
- adding testing and reporting of surface texture during construction.

**Figure 2:**
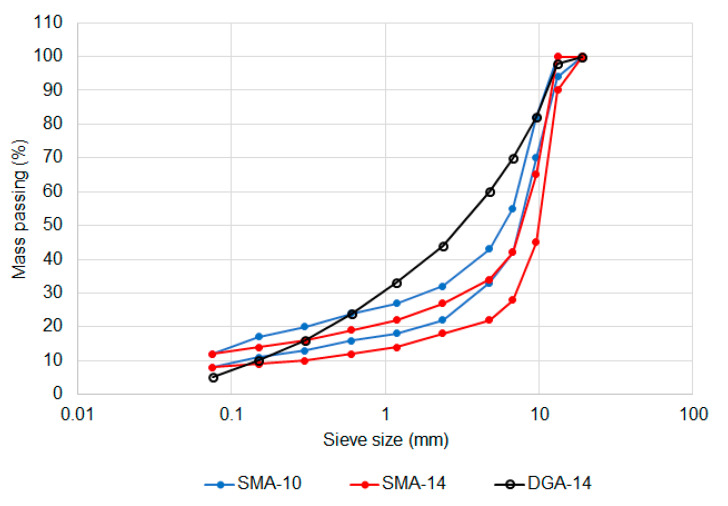
Comparison of SMA-10 and SMA-14 gradation limits.

The only change in aggregate properties was a reduction in the maximum allowable percentage of non-cubic shaped coarse aggregate particles, measured as the flakiness index. Two SMA volumetric compositions were identified as being viable, one based on German airport requirements, with a nominal maximum aggregate size of 10 mm, and the other based on Chinese airport requirements, with a nominal maximum aggregate size of 14 mm [14]. The aggregate gradations are compared in Figure 2 and the other volumetric properties are in Table 3.

Marshall-prepared samples are commonly used for both DGA and SMA in Australia. However, the 75 blows commonly used for airport DGA presents the risk of aggregate crushing when applied to SMA, so the Marshall sample preparation was reduced to 50 blows, which is consistent with Australian SMA intended for road applications. Moreover, refusal air voids density, generally performed to AS 2891.2.2, is not tested for SMA for road applications in Australia, so this requirement was removed for airport SMA. Surface texture was measured by a volumetric sand patch on the slabs prepared for wheel tracking and the binder drain down was measured at the proposed maximum asphalt production temperature, as summarised in Table 4.

Despite these changes, the performance requirements (Table 2), asphalt production tolerances and surface construction processes, field air voids content limits and general quality requirements were kept the same for SMA as they were for DGA. For laboratory mixture design purposes, the mixtures were produced to the requirements of Figure 2 and Table 3 while the performance was evaluated based on the requirements of Table 2 and Table 4.

## 3. Materials and Methods

To evaluate SMA as an ungrooved runway surfacing option, different coarse aggregate sources were selected to be representative of the diverse sources available in Australia. Each SMA mixture was tested in the laboratory against the performance requirements established for airport asphalt in Australia, as well surface texture and binder drain down.

### 3.1. Methods

First, the mixtures were designed theoretically and then produced to confirm that the volumetric requirements for gradation, binder content and air voids (Figure 2 and Table 3) were achieved. Second, the binder drain down requirements were checked (Table 4). Finally, samples were prepared for the performance tests (Table 2 and surface texture from Table 4). Because some adjustment was made to some mixtures, the process was partly iterative. For example, some mixtures initially failed to meet the binder drain down requirement. Additional fibres were added and the test repeated to confirm the requirement was achieved before other performance testing commenced. Furthermore, some secondary test parameters were measured, such as the wheel track rutting rate and the initial modulus of the fatigue beams. The primary and Australian-specific laboratory performance tests are described as follows.

#### 3.1.1. Wheel Tracking Test

Australia uses the Copper’s wheel tracker for asphalt deformation resistance (AG:PT/T231), which involves compacting two 300 mm × 300 mm× 50 mm specimens to 5 ± 1% air void using a laboratory compactor described in AG:PT/T220 [21]. The samples are then traversed by a wheel with a vertical loading of 700 ± 20 N for 10,000 passes [22]. Multiple depth measurements are taken, and the average tracking depth is recorded. In addition to rut depth, a rut depth rate is recorded as the slope of rut depth over passes from 4000 to 10,000 passes (mm/kpass). The rut depth rate is a good indicator of the secondary creep expected after the initial deformation caused by early trafficking.

For context, Austroads [23] recommends that final rut depths less than 3.5 mm, at the standard temperature of 60 °C, indicates superior performing asphalt when using AG:PT/T231 for Australian roads. For the purpose of Australian airport asphalt, a suitably performing DGA must have no greater than 2 mm rut depth at 65 °C. The higher test temperature is particular to grooved runway surfaces because the grooved (upper 6 mm only) portion of the surface is exposed to higher temperatures than the middle of the surface layer, which is more applicable to surface rutting and shoving [7].

#### 3.1.2. Fatigue Life

Brittle fracture is not an issue in the Australian climate, but intermediate temperature asphalt fatigue is assessed using repeated load four-point bending at 20 °C, according to AG:PT/T274 [24]. This method measures the initial stiffness of rectangular beams with dimensions 390 mm× 50 mm× 63.5 mm, prepared to 5 ± 0.5% air voids content. The samples are subjected to sinusoidal load at a controlled level of maximum strain magnitude of 200 µε. The fatigue life is defined as the number of cycles until the measured stiffness is reduced to 50% of the initial stiffness, although it is common to terminate the test after 500,000 load cycles and record the stiffness at that time, as a percentage reduction from the initial stiffness value. A 50% (or less) reduction in stiffness is considered good fatigue resistance and most Australian airport asphalt mixtures, which generally contain 5.4–5.8% of highly modified elastomeric binder, generally achieve this requirement with ease [25].

#### 3.1.3. Particle Loss

Australia uses the Cantabro losses test, which measures the loss of sample mass during abrasive conditioning of either gyratory or Marshall compacted asphalt samples, according to AG:PT/T236 [26]. Cantabro losses is commonly used for open graded friction course mixtures, due to the reliance on the binder cohesion and adhesion to the aggregate particles for mixture durability. The samples are weighed before and after exposure to 300 revolutions in a Los Angeles abrasion drum. Mass loss not greater than 15% of the initial sample mass is considered to represent good resistance to abrasive particle loss. However, the Cantabro losses test only assesses particle loss through abrasion and the application of mechanical energy when the asphalt sample impacts the walls of the drum. In airport asphalt, fretting and ravelling are more commonly caused by the deterioration of the binder due to oxidation, aggregate breakdown due to moisture exposure and general weathering [27]. The particle losses test does not account for this and an accelerated laboratory asphalt mastic weathering and ageing test is required [28]. However, a more suitable test is not available at this time and the particle losses test was used for this research. It has been established that airport DGA produced with polymer modified binder generally has a particle loss of less than 5%.

#### 3.1.4. Tensile Strength Ratio

Australia uses the modified Lottman test for resistance to moisture damage, also known as asphalt stripping, as detailed in test method AG:PT/T232 [29]. Six nominally identical samples are compacted to 8 ± 1% air voids in a gyratory compactor and tested for indirect tensile strength at 25 °C. Three samples are tested unconditioned and the other three are conditioned by water saturation and a freeze–thaw cycle. The tensile strength ratio (TSR) is calculated as the average of the conditioned results, divided by the average of the unconditioned results. Australia considers asphalt mixtures to have good resistance to moisture damage if the TSR exceeds 80% and many airport asphalt mixtures achieve 90% or better.

#### 3.1.5. Surface Texture

Surface macrotexture for Australian pavements is measured using a volumetric sand patch method as detailed in AG:PT/250 [30] (Austroads 2008). The method requires a known volume of fine sand to be placed on the asphalt surface and spread in a circle until the macrotexture is full and no sand remains above the surface texture level. The diameter of the sand patch is volumetrically related to the average surface texture depth. It is well established that 14-mm sized airport asphalt surfaces have a surface texture of 0.4–0.6 mm [5], while SMA and open graded surfaces can result in surface texture depths of 1.5 mm or more [31].

#### 3.1.6. Binder Drain Down

Australia uses a version of the Schellenberg test [32] to assess the risk of binder drain down of SMA mixtures, as detailed in AG:PT/T235 [33]. The method allows the binder in a 1-kg sample of asphalt to drain down at fixed temperature for a period of one hour. The asphalt is then removed from the container and the residual binder mass is calculated as a percentage of the initial mass of binder in the asphalt sample. Some jurisdictions prefer to specify a minimum fibre content, with 0.3% typically providing an acceptable result. However, for a performance-related specification, the outcome-focussed test is preferred over a prescriptive fibre content.

### 3.2. Raw Materials

Four different aggregate sources were used to produce seven SMA mixtures, three to the SMA-10 volumetric requirements and four to the SMA-14 requirements. To ensure the SMA mixtures were diverse and representative, Latite, Basalt, Amphibolite and Greywacke aggregates were selected. The aggregate sources had all been used previously, for either heavy-duty road or airport asphalt production. Consequently, they were all of a hard and durable nature, with properties suitable for airport wearing course applications. For example, all course aggregates were 100% crushed with a flakiness index below 20%.

The same elastomeric polymer modified binder (PMB) known in Australia as A15E [34] was used for the production of all seven mixtures, with the key binder properties detailed in Table 5. A PMB was selected as most airfield and heavy-duty road applications use a PMB for improved performance. A15E was chosen as it is recommended as a suitable binder for SMA applications [35] and is specified in a number of heavy-duty road applications in Australia, as well as some use in airport DGA surfacing applications.

### 3.3. Asphalt Mixtures

The performance of SMA was evaluated by comparing the various test results to the airport performance requirements (Table 2) and by considering the general trends across the four aggregate types and by comparison of the results for the Chinese-based (14 mm) and German-based (10 mm) volumetric compositions. The seven mixture designations, including their added filler type and cellulose fibre content, are summarised in Table 6. Only the 14 mm mixture was produced with the Greywacke aggregate due to material availability.

## 4. Results

**Figure 3:**
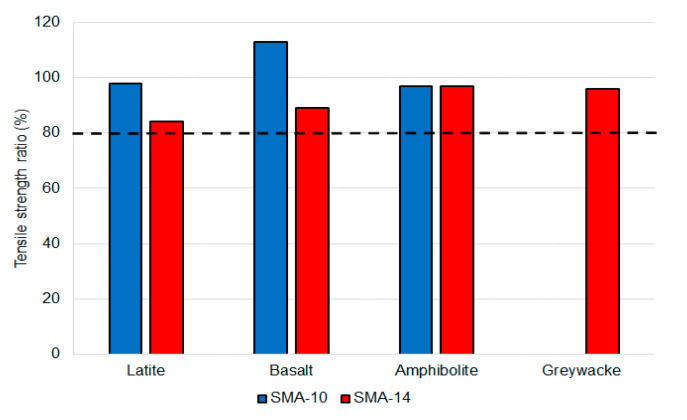
Moisture damage test results.

**Figure 4:**
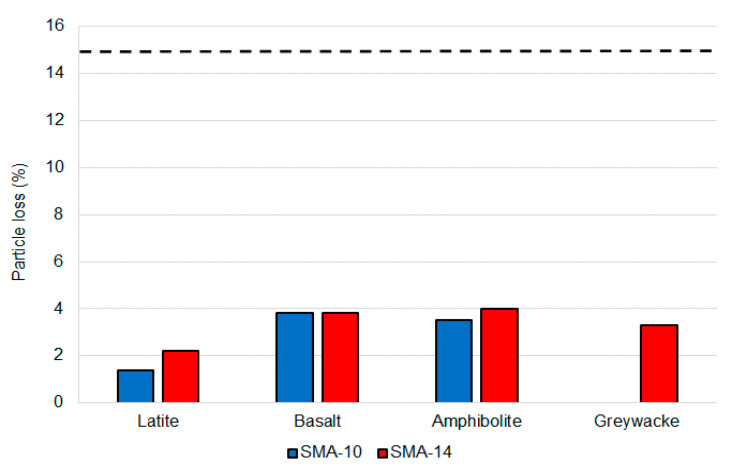
Particle loss rest results.

The gradation results are summarised in Table 7 and the other volumetric properties are in Table 8. Moisture damage results are in Figure 3 and the particle loss results are in Figure 4. The wheel tracking results are in Table 9 and the fatigue results are in Table 10. Surface texture results are in Table 11 and the binder drain down results are in Table 12. The binder drain down test was initially performed at 185 °C and then repeated at 175 °C if the first test result did not meet the requirement.

## 5. Discussion

### 5.1. Compositional Requirements

**Figure 5:**
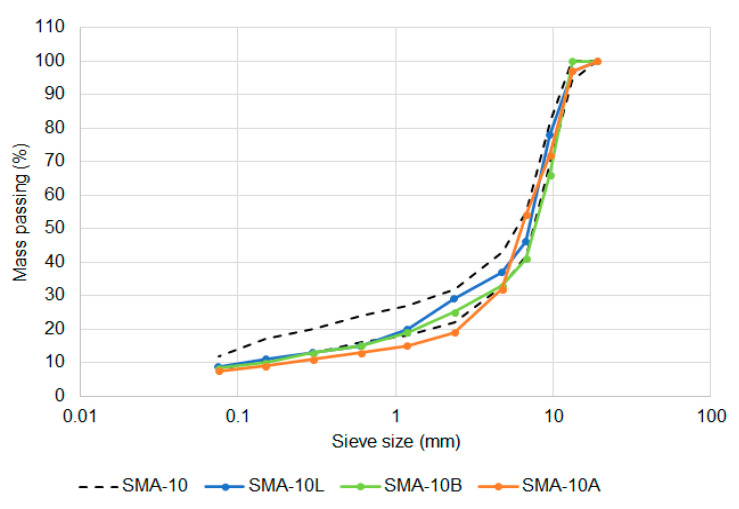
SMA-10 gradations compared to target.

**Figure 6:**
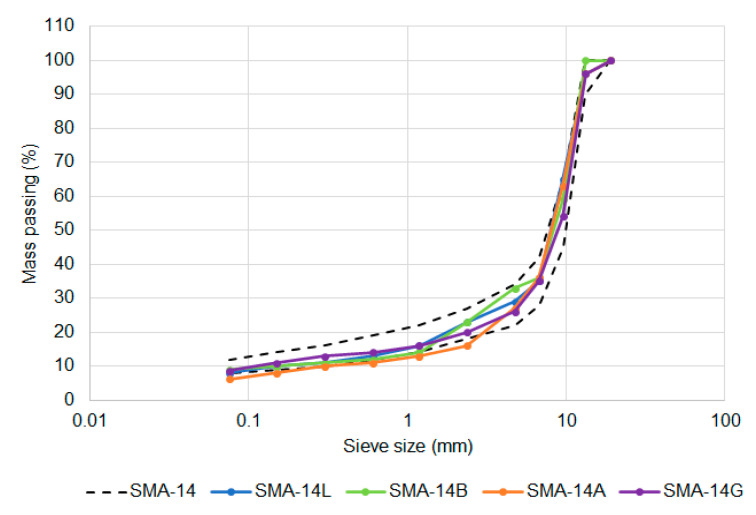
SMA-14 gradations compared to target.

Before the performance of the mixtures was considered, it was necessary to verify that the seven mixtures achieved the intended compositions. The gradations were generally on the coarser side of the target limits, with the Amphibolite-based mixture falling below the target envelope for both the 10-mm (Figure 5) and 14-mm (Figure 6) sized options. The shape of the two target gradations were similar, with the 10-mm sized mixture having an average of 4% more aggregate passing each sieve than for the equivalent 14 mm mixtures.

The smaller sized mixtures had a 1–2% lower VMA, reflecting the more closed up aggregate skeleton, and a generally 0.0–0.2% higher binder content, as implied by the target requirements (Table 3). The 10 mm mixture air void contents were also generally around 1% higher than for the 14 mm mixtures. It is noted that VMA, air voids content and binder content are mathematically related, based on the relative aggregate and binder densities.

The seven mixtures generally met the compositional requirements, within the tolerances normally expected when attempting to balance various volumetric properties with diverse aggregate sources. Consequently, it was concluded that the measured differences in the 10 mm and 14 mm SMA performance were generally representative of the typical SMA mixtures expected to be used on Australian runway surfaces.

### 5.2. Deformation Resistance

**Figure 7:**
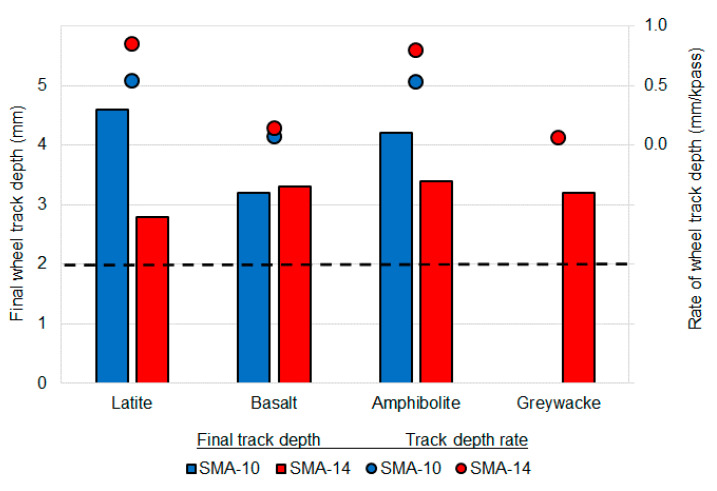
Average SMA wheel track depth and rate summary.

**Figure 8:**
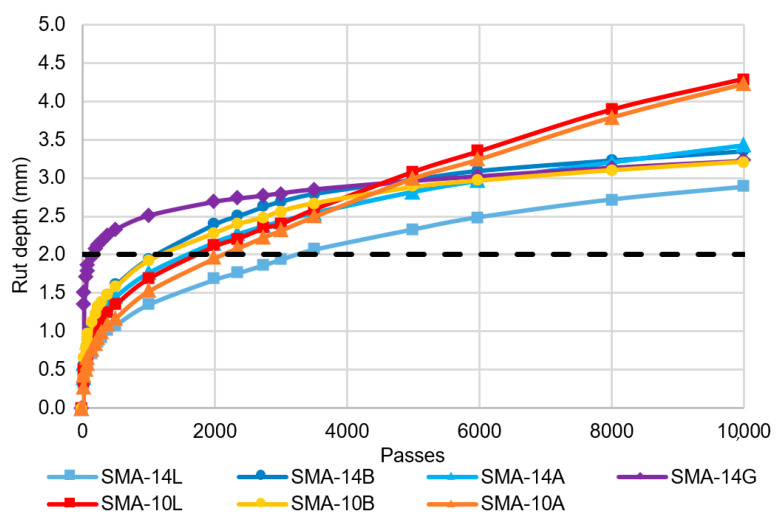
Wheel track depth growth.

All mixtures failed to meet the airport asphalt requirement for deformation resistance, measured using wheel track rutting depth (Figure 7). On average, the final rut depth associated with the 14-mm sized mixtures was 18% lower than for the 10 mm mixtures, although the difference was not statistically significant (p-value 0.27). The SMA wheel track rut depths exceeded those generally achieved for airport DGA mixtures [25] and this is not consistent with the established benefits of SMA including high deformation resistance [5]. Furthermore, the rate of growth of the wheel tracking depth varied, with some samples deforming significantly during the first 500 cycles, but further deformation then reducing significantly (e.g., SMA-14G), while other samples deformed at a more consistent rate over all 10,000 passes (e.g., SMA-14L), as shown in Figure 8.

Jamieson and White [36] demonstrated the sensitivity of asphalt wheel tracking results to the air voids content and sample test temperature. The current Australian airport asphalt specification tests wheel tracking at 65 °C on samples prepared at a target 5% air voids content. The test temperature is higher than typically used in Australia, which is 50–60 °C, and is intended to better represent the temperature of the surface grooves in the field, rather than the mid-layer temperature. However, this is not applicable to ungrooved SMA. Furthermore, to ensure DGA grooves do not close under traffic during high temperatures, more polymer modified binders are generally used. These binders have high softening points [37] and result in low wheel tracking rut depths. In contrast, airport SMA is expected to be produced with moderately polymer modified binders, such as the A15E used in this research, which have lower softening points and are therefore more susceptible to deformation at elevated temperatures. Regardless of the mixture type, the specimen target air void content is intended to reflect field air voids. Because SMA generally has around 1% lower in-service air voids than DGA, the 5% target is appropriate for DGA but not for SMA.

**Figure 9:**
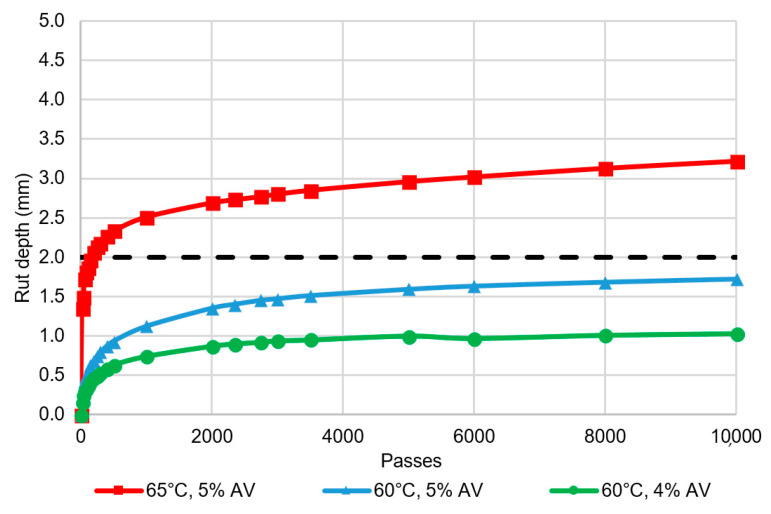
Effect of temperature and air voids on wheel track depth.

To confirm these effects, one SMA mixture (SMA-14G) was re-tested at 60 °C, at both 4% and 5% target air voids contents (Figure 9). The final rut depth reduced from 3.2 mm (65 °C and 5% air voids) to 1.0 mm (60 °C and 4% air voids). The sample air voids content had a significant effect on the initial deformation, while the test temperature had more influence on the ongoing rate of deformation. This indicates that SMA meets the airport asphalt deformation resistance requirement when tested under conditions more representative of its expected field conditions. However, practical implementation of this difference in testing requires different test conditions to be nominated for SMA and DGA mixture designs in the future.

### 5.3. Fatigue Resistance

**Figure 10:**
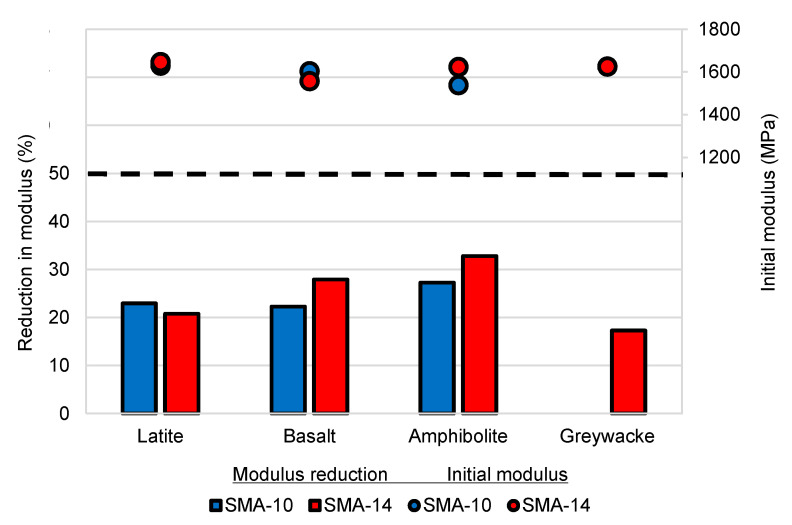
Average SMA reduction in modulus in fatigue and initial modulus summary.

All mixtures exceeded the fatigue resistance requirements, with reductions in modulus of 17–33% (Figure 10). This is comparable to airport DGA performance and reflects the relatively high binder content used in both DGA and SMA for airports, as well as the elastomeric modified binder used. On average, the 14 mm mixtures had a 12% lower reduction in modulus than the 10 mm mixtures; however, this difference was not statistically significant (*p*-value 0.37). It is also noted that the average initial modulus values were all consistent, ranging from 1540 to 1650 MPa. This is lower than typical airport DGA in Australia and this again reflects the higher content of only moderately modified binder used, compared to typical airport DGA mixtures. The SMA mixtures clearly achieved the airport asphalt performance requirement for fatigue resistance.

### 5.4. Mixture Durability

All seven SMA mixtures exceeded the TSR minimum of 80%, with TSR values ranging from 84% to 113%. These values are comparable to typical results for airport DGA produced with polymer modified binders. Similarly, the particle loss values ranged from 1.4% to 3.8%, which are all well below the 15% maximum and less than the typical 5% maximum value measured on airport DGA mixtures produced with polymer modified binders. Consequently, SMA met the durability requirements for airport asphalt surfacing mixtures, with performance like the DGA mixtures commonly used on Australian runways in the past. However, the available test methods did not include an assessment of the relative DGA and SMA binder and mastic erosion-based durability.

### 5.5. Surface Texture

**Figure 11:**
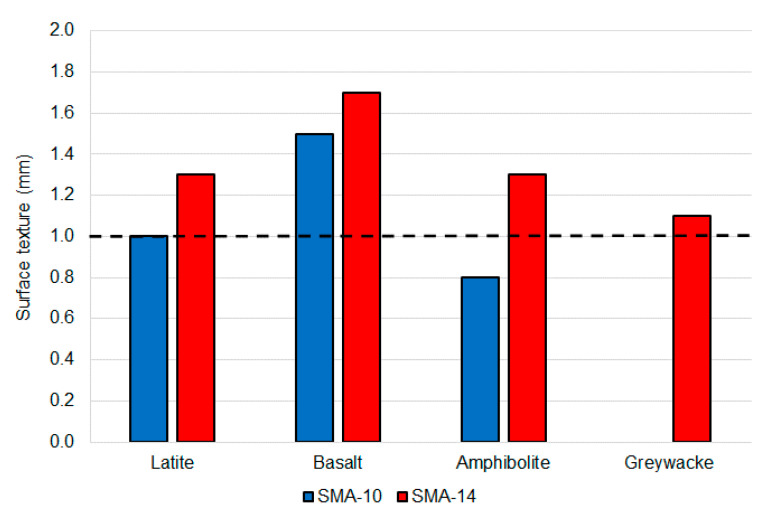
Average SMA surface texture summary.

All four of the 14 mm-sized mixtures exceeded the 1 mm surface texture required to allow grooving to be avoided under aircraft skid resistance regulations. In contrast, only one (of three) 10-mm sized mixture exceeded the 1 mm minimum (Figure 11). On average, the surface texture associated with the 14-mm sized mixtures was 35% higher than for the 10 mm mixtures and this difference was statistically significant (*p*-value 0.03). This indicates that the Chinese-based 14 mm SMA option is likely to more reliably achieve the surface texture required for SMA to be used as a ungrooved runway surface, across a broad range of aggregate types. The 10 mm option may achieve 1 mm with some aggregates, but this is expected to require targeting the coarser side of the gradation envelope during mixture design.

### 5.6. Binder Drain Down

**Figure 12:**
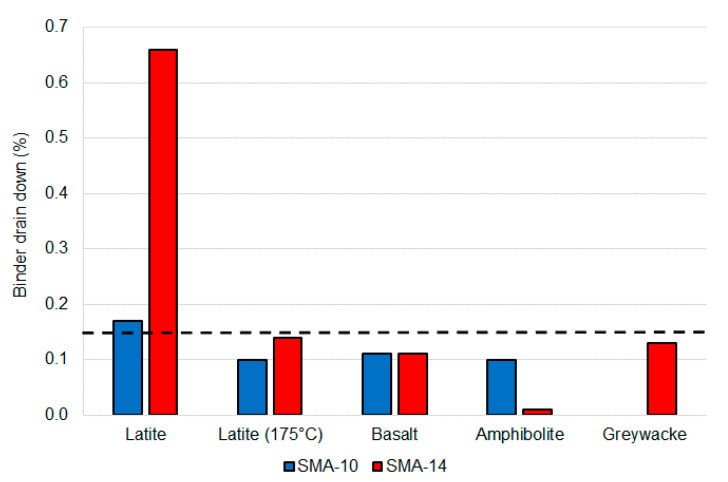
SMA binder drain down at 185 °C (except where noted) summary.

All mixtures achieved the binder drain down requirement at 185 °C, except for the two mixtures produced with the Latite aggregate. The Latite mixture fibre content was increased from 0.3% to 0.4% but the binder drain down still exceeded the 0.15% maximum at 185 °C (Figure 12). However, when retested at 175 °C, the Latite mixture achieved the requirements. This highlights the high sensitivity of binder drain down risk to asphalt production temperature, which is also likely to be affected by the binder type. It also demonstrates that a performance-related binder drain down limit is more appropriate than a minimum fibre content, which is commonly adopted by many jurisdictions [5].

## 6. Conclusions

Based on the results of seven SMA mixtures of two different maximum particle sizes and using four different aggregate sources, it was concluded that SMA generally achieved the airport asphalt performance requirements, as summarised in Table 13. It was also concluded that the 14-mm sized mixture exceeded the performance of the 10 mm mixture and that the 14 mm is preferred for implementation as an ungrooved runway surface in Australia. Additional research is required to develop an accelerated laboratory ageing test that is representative of binder oxidation and mastic erosion. Furthermore, different test protocols are required for wheel track testing as an indicator of SMA and DGA deformation resistance. It is recommended that Australian airports consider SMA-14 as an ungrooved runway surface in the future, subject to field trials and sound performance over time.

## Figures and Tables

**Table 1:** Performance requirements and standard tests for airport asphalt [2].

Requirement	Protects Against	Standard Laboratory Test
Deformation resistance	Groove closure Rutting Shearing/shoving	Wheel tracking (AG:PT/T231) Refusal Density (AS/NZS 2891.2.2)
Fracture Resistance	Top-down cracking Fatigue cracking	Fatigue Life (AG:PT/T274)
Durability	Pavement generated loose material Resistance to moisture damage	Particle loss (AG:PT/T236) Tensile Strength Ratio (AG:PT/T232)
Surface friction and texture	Skid resistance Compliance requirement	-

**Table 2:** Performance-related airport asphalt requirements [7].

Test Property	Test Method	Requirement
Indirect Tensile Strength Ratio (TSR)	AG:PT/T232	Not less than 80%
Wheel Tracking (10,000 passes at 65 °C)	AG:PT/T231	Not more than 2.0 mm
Fatigue life (at 20 °C and 200 µm)	AG:PT/T274	Not less than 500,000 cycles to 50% of initial flexural stiffness
Particle loss	AG:PT/T236	Not more than 15%

**Table 3:** SMA additional volumetric and related requirements [14].

Property	Test Method	SMA-10	SMA-14
Binder Content (% by mass)	AS/NZS 289.1.3	≥6.1	≥5.5
Marshall Air Voids (% by volume)	AS/NZS 2891.8 AS/NZS 2891.9.2 AS/NZS 289.1.7.1	2.0–4.0	3.0–5.0

**Table 4:** SMA additional performance requirements [14].

Property	Test Method	SMA-10	SMA-14
Binder drain down (% by mass)	AG:PT/T235	≤0.15	≤0.15
Surface texture (mm)	AG:PT/T250	≥1 mm	≥1 mm

**Table 5:** Properties of A15E polymer modified binder [34].

Binder Property	Test Method	Requirement
Viscosity at 165 °C (Pa·s) max	AS/NZS 2341.4	0.9
Torsional recovery at 25 °C, 30 s (%)	AG:PT/T122	55–80
Softening point (°C)	AG:PT/T131	82–105
Consistency 6% at 60 °C (Pa·s) min	AG:PT/T121	900
Stiffness at 25 °C (kPa) max	AG:PT/T121	30

**Table 6:** SMA mixture designations and details.

Designation	Aggregate	Added Filler	Fiber Content (% by Mass)
SMA-10L	Latite	Ground limestone	0.4
SMA-14L	Latite	Ground limestone	0.4
SMA-10B	Basalt	Ground limestone/hydrated lime blend	0.4
SMA-14B	Basalt	Ground limestone/hydrated lime blend	0.5
SMA-10A	Amphibolite	Dolerite baghouse fines	0.3
SMA-14A	Amphibolite	Dolerite baghouse fines	0.3
SMA-14G	Greywacke	Ground limestone	0.3

**Table 7:** SMA mixture gradation results.

Sieve Size (mm)	Percent Passing by Mass (%)						
	SMA-10L	SMA-14L	SMA-10B	SMA-14B	SMA-10A	SMA-14A	SMA-14G
19	100	100	100	100	100	100	100
13.2	97	96	100	100	97	96	96
9.5	78	65	66	59	72	63	54
6.7	46	35	41	36	54	36	35
4.75	37	29	33	33	32	27	26
2.36	29	23	25	23	19	16	20
1.18	20	16	19	14	15	13	16
0.6	15	13	15	12	13	11	14
0.3	13	11	13	11	11	10	13
0.15	11	10	10	10	9	8	11
0.075	8.6	8.0	8.3	9	7.6	6.2	8.7

**Table 8:** SMA mixture other volumetric results.

Property	SMA-10L	SMA-14L	SMA-10B	SMA-14B	SMA-10A	SMA-14A	SMA-14G
VMA (% by volume)	16	18	17	19	17	18	17
Binder Content (% by mass)	6.7	6.6	6.4	6.3	6.1	6.4	6.1
Rigden Voids (% by volume)	34	34	40	40	34	34	37
Marshall Air Voids (% by volume)	3.0	3.8	2.5	3.9	3.3	4.0	4.2

**Table 9:** Laboratory wheel tracking results (duplicate).

Aggregate	SMA-10			SMA-14		
	Air Voids (%)	Rut Depth (mm)	Tracking Rate (mm/kpass)	Air Voids (%)	Rut Depth (mm)	Tracking Rate (mm/kpass)
Latite	4.3	4.3	0.52	4.1	3.6	0.38
	4.2	5.0	0.55	4.2	2.1	0.25
Basalt	3.5	2.6	0.06	2.6	2.6	0.05
	5.2	3.8	0.08	5.6	4.1	0.09
Amphibolite	5.4	4.5	0.67	4.6	3.7	0.28
	4.1	3.9	0.38	4.1	3.2	0.23
Greywacke	-	-	-	5.1	3.1	0.06
	-	-	-	4.9	2.9	0.06

**Table 10:** Laboratory fatigue results (triplicate).

Aggregate	SMA-10			SMA-14		
	Air Voids (%)	Initial Modulus (MPa)	Modulus after 500,000 Cycles (MPa)	Air Voids (%)	Initial Modulus (MPa)	Modulus after 500,000 Cycles (MPa)
Latite	4.8	1810	1220	4.8	1614	1387
	4.8	1667	1413	5.0	1624	1222
	4.7	1413	1135	4.8	1702	1305
Basalt	7.2	1637	1230	7.4	1562	1151
	7.3	1571	1237	7.5	1522	1126
	7.2	1602	1273	7.2	1588	1091
Amphibolite	5.0	1513	1110	4.6	1700	1268
	5.4	1535	1153	4.5	1760	1038
	4.6	1568	1095	5.1	1410	967
Greywacke	-	-	-	5.5	1629	1373
	-	-	-	5.5	1647	1316
	-	-	-	5.2	1601	1345

**Table 11:** Surface texture results (duplicate) (mm).

Aggregate	SMA-10		SMA-14	
Latite	1.1	0.9	1.2	1.4
Basalt	1.5	1.4	1.6	1.8
Amphibolite	0.8	0.8	1.3	1.2
Greywacke	-	-	1.0	1.1

**Table 12:** Binder drain down results.

Aggregate	SMA-10		SMA-14	
	185 °C	175 °C	185 °C	175 °C
Latite	0.17	0.10	0.66	0.14
Basalt	0.11	*	0.11	*
Amphibolite	0.10	*	0.01	*
Greywacke	-	-	0.13	*

* not tested due to acceptable results at 185 °C.

**Table 13:** Summary of SMA performance compared to airport requirements.

Requirement	SMA-10	SMA-14
Deformation resistance	Achieved when tested at conditions representative of in-service conditions	Achieved when tested at conditions representative of in-service conditions
Fracture Resistance	Achieved	Achieved
Durability	Achieved	Achieved
Surface texture	Not achieved	Achieved
Binder drain down risk	Achieved with aggregate-specific caution	Achieved with aggregate-specific caution

## Data Availability

The data presented in this study are available in Table 11 and Table 12, and Figure 3 and Figure 4.
